# The ventilatory response pattern to exercise across the age‐span in healthy adults

**DOI:** 10.14814/phy2.71065

**Published:** 2026-09-02

**Authors:** Zander J. Williams, Giovanni Cenerini, Fabian Schwendinger, Raphael Knaier, Jonathan Wagner, James H. Hull, Arno Schmidt‐Trucksäss

**Affiliations:** ^1^ Department of Respiratory Medicine Royal Brompton Hospital London UK; ^2^ Department of Surgical, Medical and Molecular Pathology and Critical Care Medicine University of Pisa Pisa Italy; ^3^ Division of Sports and Exercise Medicine, Department of Sport, Exercise and Health University of Basel Basel Switzerland; ^4^ Division of Applied Exercise Physiology, Department of Sport, Exercise and Health University of Basel Basel Switzerland; ^5^ Institute of Sport, Exercise and Health (ISEH), Division of Surgery and Interventional Science University College London London UK; ^6^ Department of Clinical Research University Hospital Basel, University of Basel Basel Switzerland

**Keywords:** dyspnea, entropy, exercise test, healthy aging, respiratory rate

## Abstract

Healthy aging is associated with a progressive decline in respiratory function and altered ventilatory mechanics, typically characterized on static physiological measurements. We aimed to evaluate the ventilatory response to exhaustive exercise across the age‐span and hypothesized that aging would be associated with inefficient alterations that may be relevant in the etiology of breathlessness. We analyzed ventilatory data obtained from cardiopulmonary exercise tests performed in 526 healthy adults (aged 20–91 years; 52% female) from the COmPLETE cohort. Sample Entropy (SampEn) was used to quantify the irregularity of minute ventilation (V̇_E_), breathing frequency (Bf), and tidal volume (V_T_) time series. Age versus entropy relationships were assessed using penalized cubic splines, adjusting for sex, BMI, peak oxygen uptake, and FEV_1_. As a result, older age was associated with a progressive decline in peak oxygen uptake, V̇_E_, Bf, and V_T_ (all *p* < 0.001). Ventilatory efficiency also decreased across the age span, evidenced by an increased V_E_/VCO_2_ nadir, and older adults demonstrated a tachypneic breathing pattern, with a higher Bf to V̇_E_ ratio (*p* < 0.001). Contrary to our hypothesis, ventilatory SampEn remained stable through early adulthood but increased after the sixth decade (SampEn Bf *p* = 0.023; SampEn V_T_: *p* = 0.004; SampEn V̇_E_: *p* = 0.001). In conclusion, older age is accompanied by a shift in the ventilatory response to exercise, characterized by a mechanically constrained, frequency‐dominant breathing pattern and diminished ventilatory efficiency. This is associated with increases in ventilatory irregularity that become most apparent after the sixth decade. Further work is needed to determine how these alterations relate to exertional breathlessness.

## 1 INTRODUCTION

Senescence is typically associated with a deterioration in pulmonary function (Sharma & Goodwin, 2006), reduced functional capacity and exertional breathlessness (Higbee et al., 2022). The latter is increasingly evident after age 60 years, and it is estimated that approximately one quarter of people over the age of 65 report this symptom, in the absence of any cardiopulmonary disease (Ramalho et al., 2019).

Healthy aging is associated with a decline in respiratory muscle strength, loss of lung elasticity, impaired ventilatory drive, and dysregulated cardiopulmonary interaction, potentially resulting in an increasingly inefficient ventilatory response to physical activity (Sylvester et al., 2022; Turner et al., 1968). During exercise, as lung volume declines with age, individuals breathe at a higher tidal volume (V_T_) to forced vital capacity (FVC) ratio and there is a shift towards a relatively tachypneic response (i.e. shallower and faster pattern), to compensate for the altered lung mechanics (Weavil et al., 2022). This may be relevant in the etiology of exertional breathlessness, particularly as a strong association between breathing frequency (Bf) and the rating of perceived exertion during exercise has been demonstrated (Nicolò et al., 2016; Nicolò & Sacchetti, 2022).

The irregularity (or complexity) of a biological signal is increasingly recognized as a marker of health. Non‐linear statistical methods have now been used in both cardiovascular and neurological science to evaluate physiological ‘health’ (Almeida‐Santos et al., 2016; Manor & Lipsitz, 2013) A common example is the evaluation of high heart rate variability, where reduced variability of this signal may be indicative of ‘dysfunction’ and is often seen in the presence of disease (Almeida‐Santos et al., 2016).

One method of evaluating irregularity in a time‐series is to calculate the sample entropy (SampEn) of a dataset (Pincus, 1991). In this context, a highly regular, repetitive pattern has low SampEn, whilst a more complex and irregular pattern results in a higher SampEn. To date, there has been relatively limited application of SampEn to assess the irregularity of the ventilatory pattern response to exercise, but further use of this method may provide insight in the presence of breathlessness in older adults. Whilst the application of similar approaches has been applied to characterize breathing patterns in specific disease (Bansal et al., 2018), little is known regarding alterations in SampEn ventilatory response to exercise, across the adult age‐span.

In this study, we aimed to characterize how respiratory pattern and irregularity during exercise changes across the adult age‐span. Using data from the functional aging and heart failure: the COmPLETE study (Wagner et al., 2019), we describe ventilatory patterns during an exhaustive exercise bout and apply SampEn analyses to describe ventilatory irregularity. We hypothesized that older adults would have a relatively more tachypneic and constrained (i.e. lower SampEn) ventilatory response to exercise (i.e. a reduced irregularity) when compared with younger age groups.

## 2 MATERIALS AND METHODS

### 2.1 Study population and design

This was a retrospective analysis of data from healthy participants, free from cardiopulmonary disease, enrolled in the COmPLETE cohort study, conducted in collaboration between the Royal Brompton Hospital, London, and the University of Basel, Switzerland.

The COmPLETE cohort study was a cross‐sectional, single center study performed at the Department of Sport, Exercise and Health, University of Basel, Switzerland, funded by the Swiss National Science Foundation (grant 182,815) and approved by the Ethics Committee of Northwestern and Central Switzerland (EKNZ 2017–01451). Written informed consent was obtained from all participants before the start of the study and performed in accordance with the Declaration of Helsinki. Detailed recruitment procedure and the full list of inclusion and exclusion criteria are described previously, including being free from an active respiratory infection (Wagner et al., 2019). Participants underwent physiological investigations, including cardiopulmonary exercise testing (CPET) on a cycle ergometer (Wagner et al., 2019, 2021).

### 2.2 Study procedures and data processing

#### 2.2.1 Cardiopulmonary exercise testing and pulmonary function

Spirometry was interpreted using the European Respiratory Society (ERS) and American Thoracic Society (ATS) guidelines (Miller et al., 2005), with race neutral Global Lung Initiative (GLI) values applied to determine percentage predicted values (Moffett et al., 2023). Maximal voluntary ventilation (MVV_est_) was estimated by multiplying the forced expiratory volume in 1 s (FEV_1_) by a factor of 40 (Wagner et al., 2019). The ratio of peak exercise minute ventilation (V̇_E_) to MVV_est_ was used as a marker of ventilatory constraint (ventilatory reserve utilization). The ratio of Bf to minute ventilation (Bf/V̇_E)_ was used as a measure of tachypneic pattern.

Cardiopulmonary exercise testing was performed in accordance with American Heart Association guidelines and as described previously (Balady et al., 2010; Wagner et al., 2019). Cardiopulmonary and ventilatory data were acquired on a breath‐by‐breath basis and averaged over 10‐s intervals. Two independent experts identified the first ventilatory threshold (VT1) using the nadir of the V̇_E_ equivalent for oxygen together with the point at which end tidal oxygen began to rise. It was then corroborated using the V‐slope method as previously described (Beaver et al., 1986; Wagner et al., 2019).

#### 2.2.2 Calculation of ventilatory regularity by entropy

The irregularity of exercise ventilation was quantified by calculating SampEn of the 10‐s average data of V̇_E_, Bf, and V_T_ time series, using the R‐package ‘TSEntropies’ v0.9 (Pincus et al., 1991; Richman & Moorman, 2000). Sample entropy was calculated as follows:

$$\text{SampEn}\left(m,r,N\right)=-\ln\left(A/B\right)$$

where *B* is the number of template vector pairs of length *m* that match within tolerance *r* × SD of the time series, and *A* is the number of template vector pairs of length *m* + 1 that also match within the same tolerance (Richman & Moorman, 2000). For each participant's time‐series, SampEn was calculated using an embedding dimension of *m* = 2 and a tolerance coefficient of *r* = 0.2. The number of data points (*N*) per participant, corresponding to the number of 10‐s intervals during the exercise test, ranged from 36 to 108, with a mean (SD) of 62.6 (14.7).

### 2.3 Statistical analysis

All statistical analyses were performed using R software (Version 4.5.0). Descriptive statistics were generated to characterize the study population, with continuous variables presented as mean (standard deviation) or median (interquartile range) as a non‐parametric equivalent, and categorical variables as *n* (%), stratified by age decade.

The primary and secondary analyses were conducted using Generalized Additive Models (GAMs) to accommodate non‐linear relationships. The relationship between ventilatory entropy measures and age was assessed using GAMs. Age was modeled as a non‐linear term using a penalized cubic spline, and models were adjusted for sex, body mass index (BMI), body mass adjusted V̇O_2peak_, FEV_1_, and exercise duration. As a secondary analysis, an age‐by‐sex interaction term was included in the GAMs to assess if the relationship between age and entropy measures differed by sex. The relationship between ventilatory entropy and body mass adjusted V̇O_2peak_ was also assessed, adjusting for age, sex, and BMI. A *p*‐value <0.05 was considered statistically significant for all analyses (Wasserstein & Lazar, 2016).

## 3 RESULTS

### 3.1 Participant characteristics

A total of 526 healthy adults (52% female), aged 20 to 91 years, were included in the analysis. Participant numbers, sex distribution and BMI were similar across age decades.

As expected, all participants had a normal mean (SD) BMI (23.7 kg/m^2^ [2.7]), FEV_1_ percent predicted (107% [22]), and FVC percent predicted (110% [22]). Absolute FEV_1_ and FVC values declined progressively with age (Table S1).

### 3.2 Cardiopulmonary exercise test findings at ventilatory threshold 1

**FIGURE 1:**
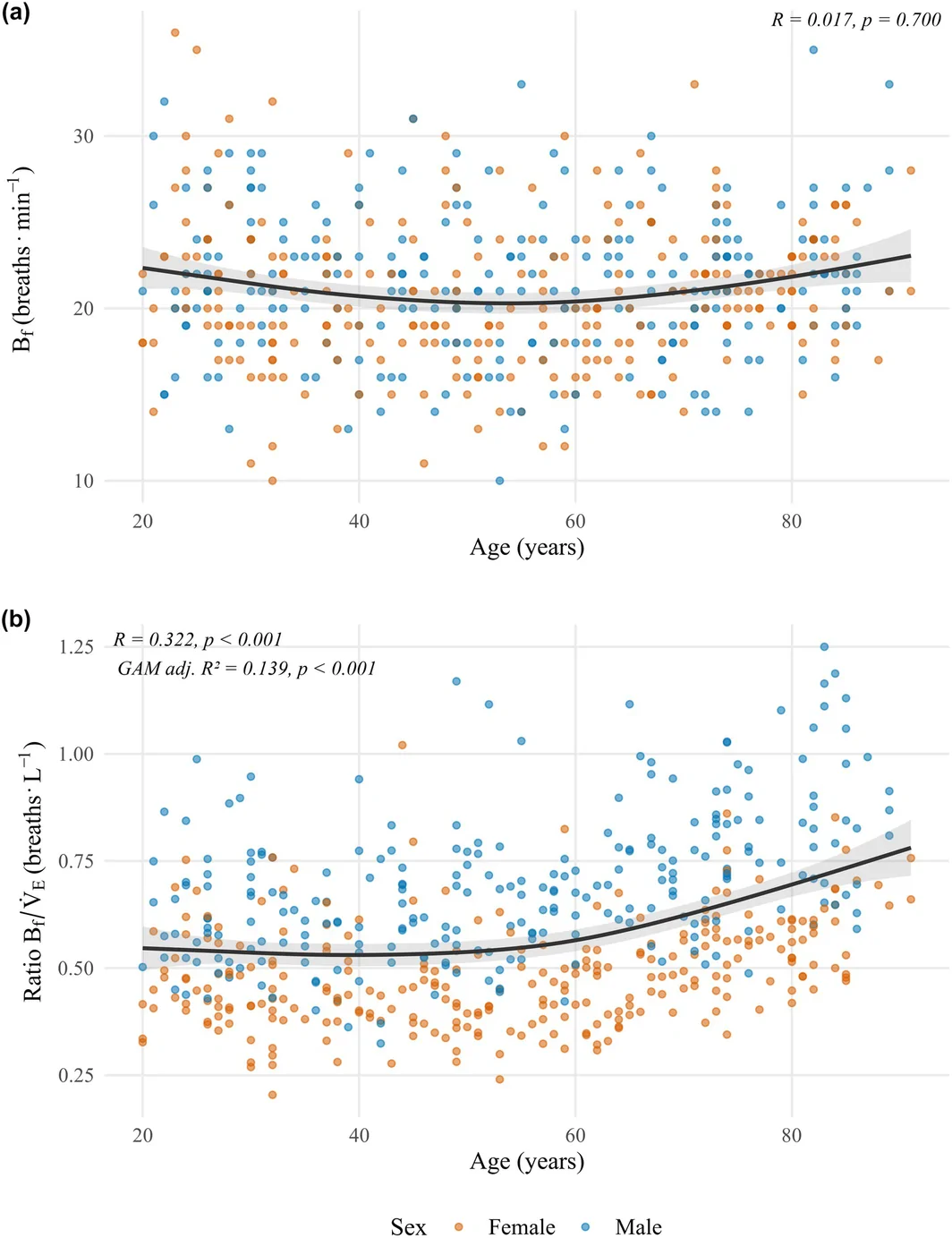
Scatter plots displaying the relationship between age and (a) Bf at VT1; and (b) Bf to ratio V̇_E_ ratio. Participants data is represented as light blue data points, with solid black lines representing linear regression models the shaded area as the 95% confidence intervals. Scatter plots are displayed with alpha = 0.45 to visualize overlapping data points.

At VT1, the majority of cardiopulmonary and ventilatory parameters declined across the age span (Table 1), however, Bf remained stable (*R* = 0.017, *p* = 0.7; Figure 1a). We found a non‐linear trajectory in the ratio of Bf to V̇_E_ (Bf/V̇_E_ ratio), with a relatively stable relationship from age 20 to 55 before an upward inflection (GAM adjusted *R* ^2^ = 0.139, effective degrees of freedom [edf] = 3.03, *p* < 0.001; Figure 1b).

**TABLE 1:** Cardiopulmonary exercise test performance at ventilatory threshold 1.

	Overall (*N* = 526)	20–29 (*N* = 73)	30–39 (*N* = 79)	40–49 (*N* = 78)	50–59 (*N* = 74)	60–69 (*N* = 77)	70–79 (N = 79)	80+ (*N* = 66)
Power (W)	92 (88, 97)	122 (110, 135)	119 (110, 126)	109 (98, 127)	93 (85, 101)	78 (67, 94)	61 (53, 75)	52 (39, 57)
HR (bpm)	123 (121, 126)	144 (138, 149)	136 (131, 141)	125 (121, 130)	124 (120, 129)	117 (112, 121)	109 (105, 115)	106 (101, 113)
HR percent predicted	74 (73, 75)	73 (71, 76)	73 (70, 75)	72 (69, 73)	75 (72, 78)	75 (73, 78)	75 (73, 78.)	76 (73, 82)
V̇O_2_ percent of peak	61 (60, 62)	57 (55, 59)	58 (55, 60)	58 (56, 60)	59 (57, 61)	63 (60, 65)	65 (63, 67)	70 (66, 71)
V̇_E_ (L/min^−1^)	37 (36, 38)	44 (37, 47)	41 (38, 43)	41 (38, 44)	38 (35, 41)	37 (35, 41)	33 (31, 36)	33 (31, 35)
V̇_E_/MVV_est_ (%)	30 (29, 31)	29 (23, 37)	26 (25, 27)	27 (25, 29)	28 (26, 30)	29 (27, 31)	33 (31, 35)	34 (32, 36)
Bf (breaths/min)	21 (21, 21)	21 (18, 23)	21 (20, 22)	20 (19, 22)	20 (19, 22)	20 (18, 22)	21 (20, 22)	22 (21, 22)
Bf/V̇_E_ Ratio (%)	56 (54, 58)	50 (42, 59)	52 (49, 56)	51 (45, 58)	51 (47, 57)	51 (49, 57)	61 (55, 65)	67 (62, 71)
V_T_ (L)	1.8 (1.7, 1.9)	2.0 (1.7, 2.2)	1.9 (1.8, 2.0)	2.0 (1.8, 2.2)	1.9 (1.8, 2.2)	2.0 (1.8, 2.1)	1.6 (1.5, 1.8)	1.5 (1.4, 1.6)
V_T_/FVC Ratio (%)	45 (44, 46)	37 (36, 46)	41 (40, 44)	45 (39, 47)	44 (41, 46)	48 (45, 50)	47 (45, 50)	48 (46, 51)
V̇_E_/V̇CO_2_ nadir	26 (25, 26)	24 (22, 25)	24 (23, 25)	25 (24, 26)	25 (25, 26)	27 (27, 28)	29 (27, 30)	31 (30, 32)
RER	0.91 (0.90, 0.91)	0.93 (0.92, 0.94)	0.93 (0.91, 0.94)	0.92 (0.90, 0.93)	0.92 (0.91, 0.93)	0.91 (0.90, 0.93)	0.87 (0.85, 0.88)	0.86 (0.85, 0.89)
P_ET_CO_2_ (mmHg)	41 (40, 41)	44 (43, 45)	43 (42, 44)	41 (41, 43)	42 (42, 43)	40 (39, 40)	38 (37, 39)	36 (35, 37)

*Note*: Values presented as Median (Q1, Q3).

The percentage of peak V̇O_2_ at VT1 increased with age (58% in the 20–29 group vs. 69% in the 80+ group; *R* = 0.37, *p* < 0.001), but the absolute time spent above VT1 was not associated with age (*R* = −0.066, *p* = 0.13). The percentage of test duration above VT1 was similar across all age decades (range: 52–54%; *R* = 0.079, *p* = 0.07).

### 3.3 Cardiopulmonary exercise test findings at peak exercise

There was a progressive reduction in exercise capacity, gas exchange, and ventilatory measures at peak exercise across the age‐span (Table 2).

**TABLE 2:** Cardiopulmonary exercise test performance at peak exercise.

	Overall (*N* = 526)	20–29 (*N* = 73)	30–39 (*N* = 79)	40–49 (*N* = 78)	50–59 (*N* = 74)	60–69 (*N* = 77)	70–79 (*N* = 79)	80+ (*N* = 66)
Power (W)	197 (142, 258)	249 (199, 315)	258 (204, 295)	230 (195, 279)	200 (169, 262)	184 (128, 244)	141 (100, 179)	100 (75, 139)
HR (bpm)	173 (158, 186)	192 (184, 196)	187 (181, 192)	178 (172, 187)	172 (165, 182)	168 (151, 175)	158 (146, 163)	147 (127, 154)
HR percent predicted	103 (97, 109)	98 (95, 101)	101 (97, 104)	103 (98, 106)	105 (100, 109)	107 (98, 113)	108 (101, 112)	106 (93, 113)
V̇O_2_ (mL/kg^−1^/min^−1^)	34.5 (26.8, 42.4)	44.8 (38.9, 48.7)	42.4 (36.9, 47.0)	38.4 (33.3, 45.5)	34.3 (30.0, 40.3)	30.5 (25.7, 38.1)	26.9 (23.1, 31.5)	22.7 (19.4, 25.8)
V̇_E_ (L/min^−1^)	102 (78, 132)	129 (100, 151)	125 (105, 144)	109 (95, 142)	103 (88, 133)	97 (74, 126)	77 (60, 107)	67 (52, 82)
V̇_E_/MVV_est_ (%)	20 (9, 30)	21 (10, 32)	21 (12, 29)	14 (5, 27)	16 (6, 30)	18 (5, 28)	19 (11, 31)	25 (14, 36)
Bf (breaths/min)	46 (40, 54)	56 (48, 60)	49 (42, 58)	49 (42, 56)	48 (42, 56)	44 (40, 50)	43 (37, 47)	39 (33, 45)
Bf/V̇_E_ Ratio (%)	46 (37, 56)	43 (37, 49)	39 (34, 46)	43 (36, 51)	44 (36, 54)	46 (38, 57)	53 (42, 65)	58 (48, 71)
V_T_ (L)	2.2 (1.8, 2.7)	2.3 (2.0, 2.7)	2.6 (2.2, 2.9)	2.3 (2.0, 2.8)	2.3 (1.9, 2.8)	2.2 (1.8, 2.6)	1.9 (1.5, 2.4)	1.7 (1.4, 2.1)
V_T_/FVC Ratio (%)	54 (48, 59)	47 (43, 54)	52 (48, 55)	52 (47, 57)	55 (49, 59)	57 (50, 61)	57 (52, 62)	59 (53, 63)
V̇_E_/V̇CO_2_ Nadir	26 (24, 29)	24 (22, 25)	24 (23, 26)	25 (24, 27)	26 (24, 27)	27 (26, 29)	30 (27, 31)	32 (30, 34)
RER	1.16 (1.11, 1.21)	1.19 (1.15, 1.23)	1.19 (1.15, 1.22)	1.18 (1.13, 1.22)	1.18 (1.15, 1.22)	1.16 (1.11, 1.21)	1.11 (1.06, 1.17)	1.07 (1.02, 1.11)
P_ET_CO_2_ (mmHg)	33 (30, 36)	34 (31, 38)	35 (31, 37)	34 (32, 36)	33 (31, 35)	32 (30, 35)	32 (30, 35)	31 (29, 34)

*Note*: Values presented as Median (Q1, Q3).

**FIGURE 2:**
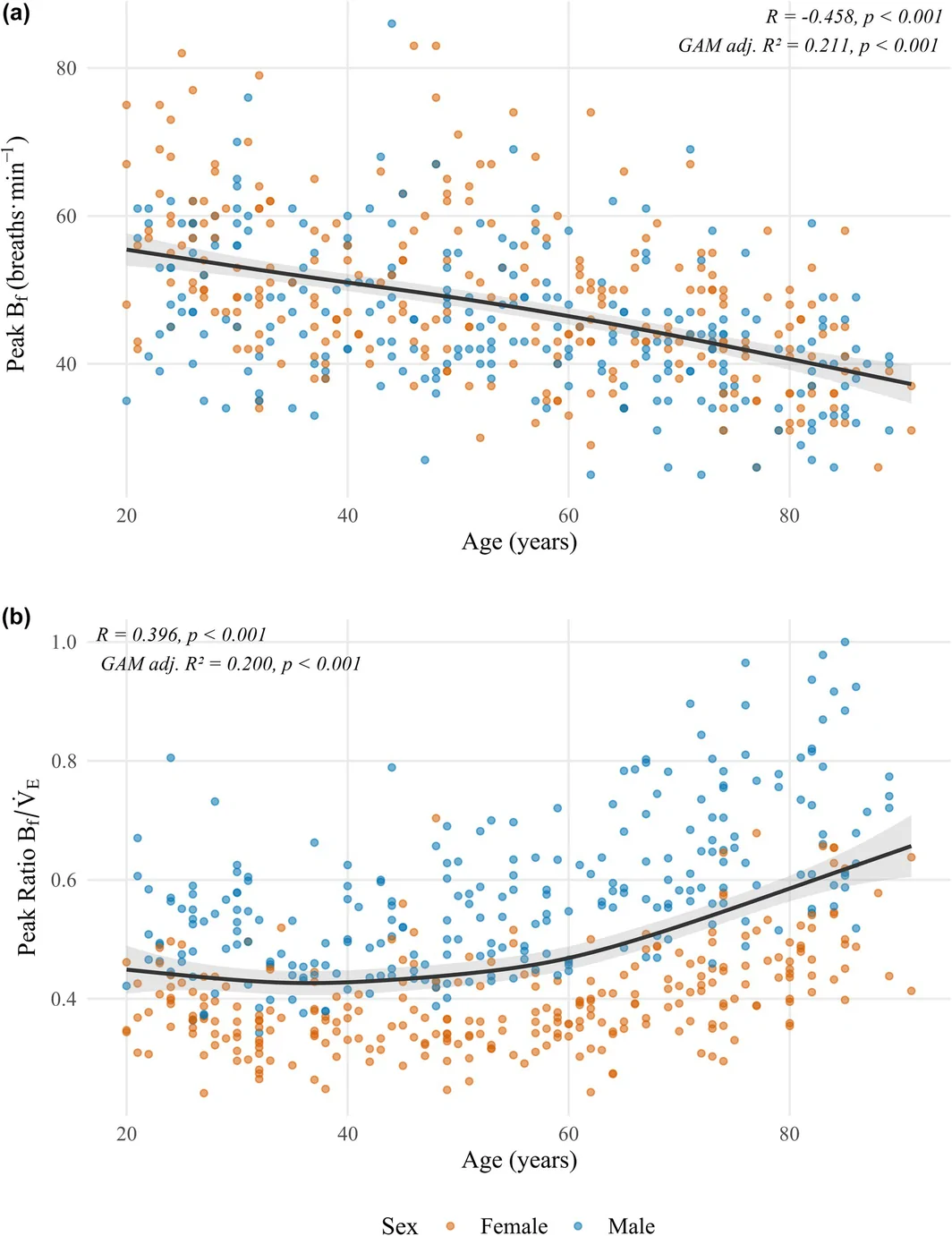
Scatter plots displaying the relationship between age and (a) Peak Bf; and (b) peak Bf to ratio V̇_E_ ratio. Participants data is represented as light blue data points, with solid black lines representing linear regression models and the shaded area the 95% confidence intervals. Scatter plots are displayed with alpha = 0.45 to visualize overlapping data points.

A negative relationship was found between age and peak exercise power (*R* = −0.62, 95% CI [−0.67, −0.56]; *p* < 0.001), V̇O_2_ (*R* = −0.69, 95% CI [−0.73, −0.64]; *p* < 0.001), V̇_E_ (*R* = −0.54, 95% CI [−0.60, −0.48]; *p* < 0.001), V_T_ (*R* = −0.36, 95% CI [−0.43, −0.28]; *p* < 0.001) and Bf (*R* = −0.46, 95%CI [−0.52, −0.39]; *p* < 0.001; Figure 2a).

#### 3.3.1 Exercise ventilatory pattern, reserve and efficiency across the age‐span

**FIGURE 3:**
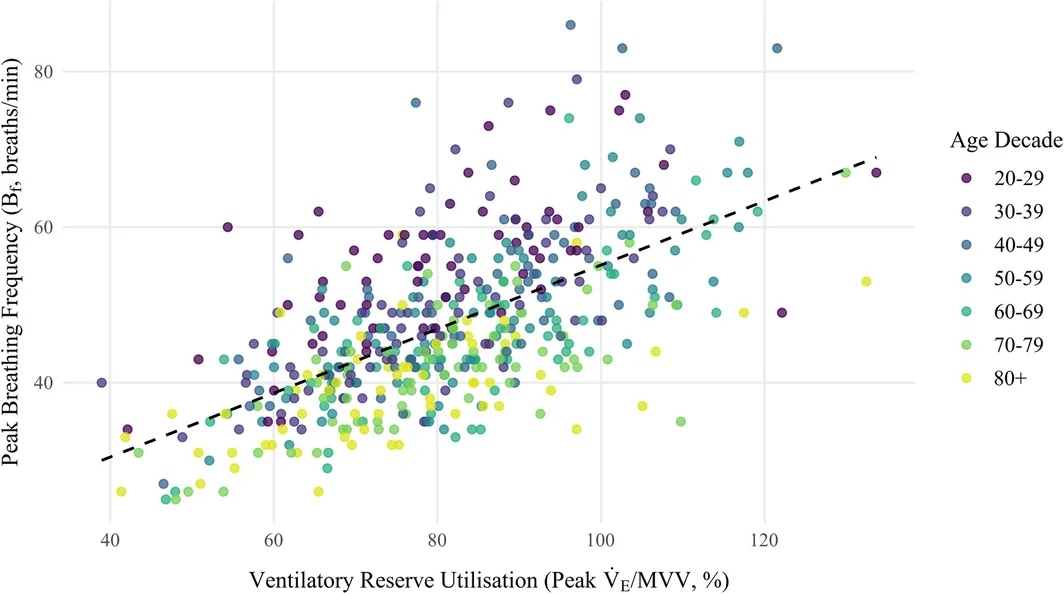
Relationship between peak exercise breathing frequency and ventilatory reserve utilization (Peak V̇_E_/MVV_est_ %). Each point represents an individual participant, with colors indicating different age decades. The dashed line represents the overall linear trend.

There was a positive relationship between Peak V_T_/FVC ratio and age (*R* = 0.40, 95% CI [0.32, 0.47], *p* < 0.001), and older adults presented with a higher Bf/V̇_E_ ratio and inflection point at around age 55–60 years (GAM adjusted, *R* ^2^ = 0.200, edf = 3.27, *p* < 0.001; Figure 2b). Multiple linear regression analysis revealed age (*β* = 0.23, 95% CI [0.17, 0.29], *p* < 0.001) and peak Bf (*β* = 1.2, 95% CI [1.0, 1.3], *p* < 0.001) were independent predictors of ventilatory reserve utilization (Table S2, Figure 3).

Univariate analysis revealed no association between age and peak V̇_E_/MVV_est_ ratio (*R* = −0.06, *p* = 0.18).

Ventilatory efficiency declined across the age‐span, as evidenced by a progressive increase in the V̇_E_/V̇CO_2_ nadir from a median (IQR) of 24 (Umetani et al., 1998; Wasserstein & Lazar, 2016) in the young adults group to 32 (Bellemare et al., 2003; Sleimen‐Malkoun et al., 2014) in the 80+ decade group (*p* < 0.001).

### 3.4 Ventilatory entropy across the age‐span

**FIGURE 4:**
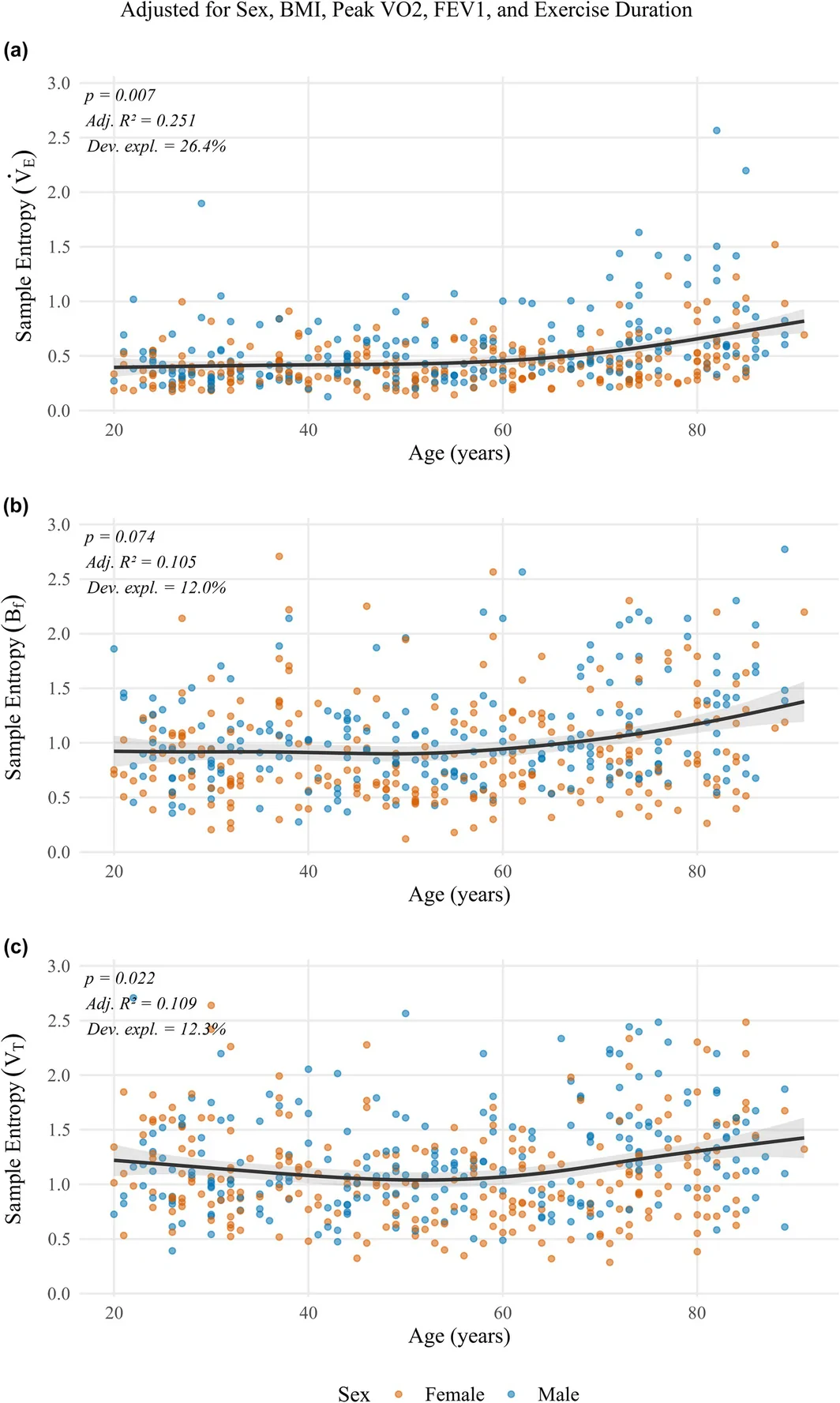
Non‐linear regression between age and sample entropy (a) V̇_E_, (b) Bf, and (c) V_T_, assessed using penalized cubic spline models. All models were adjusted for sex, BMI, V̇O_2peak_, FEV_1_, and exercise duration, with the *p*‐value for the non‐linear age term reported.

Once adjusted for sex, BMI, V̇O_2peak_, FEV_1_, and exercise duration, a non‐linear relationship was observed between age and SampEn ventilatory measures (Figure 4; Table S3). Entropy values remain stable through early adulthood and middle age before increasing progressively from approximately age 60 years onwards; a pattern consistent across SampEn V̇_E_ (adjusted *R* ^2^ = 0.251, deviance explained = 26.3%; *p* = 0.001), V_T_ (adjusted *R* ^2^ = 0.115, deviance explained = 12.9%; *p* = 0.004), and Bf (adjusted *R* ^2^ = 0.131, deviance explained = 16.6%; *p* = 0.023) (Table 3).

**TABLE 3:** Ventilatory entropy parameters by age decade.

Characteristic	Overall (*N* = 526)	20–29 (*N* = 73)	30–39 (*N* = 79)	40–49 (*N* = 78)	50–59 (*N* = 74)	60–69 (*N* = 77)	70–79 (*N* = 79)	80+ (*N* = 66)
SampEn V_T_	1.08 (0.82, 1.42)	1.13 (0.88, 1.47)	1.04 (0.86, 1.39)	0.98 (0.77, 1.23)	1.08 (0.75, 1.30)	0.90 (0.74, 1.27)	1.24 (0.92, 1.58)	1.25 (0.96, 1.58)
SampEn Bf	0.90 (0.66, 1.24)	0.90 (0.69, 1.12)	0.86 (0.66, 1.18)	0.80 (0.58, 1.06)	0.74 (0.53, 1.11)	0.93 (0.70, 1.23)	0.93 (0.72, 1.35)	1.35 (0.83, 1.61)
SampEn V̇_E_	0.42 (0.31, 0.58)	0.35 (0.26, 0.47)	0.35 (0.28, 0.45)	0.41 (0.27, 0.52)	0.37 (0.29, 0.60)	0.44 (0.36, 0.52)	0.47 (0.32, 0.69)	0.59 (0.45, 0.90)

*Note*: Values presented as Median (Q1, Q3).

SampEn V̇_E_ was greater in males compared to females (standardized *β* = 0.38; *p* = 0.004). Age‐sex interactions were present for SampEn V̇_E_ (*p* = 0.017) and SampEn V_T_ (*p* = 0.004), but not SampEn Bf (*p* = 0.6) (Table S4). An inverse relationship was found between exercise duration and SampEn V̇_E_ (*β* = −0.01, 95% CI [−0.03, 0.00], *p* = 0.010; standardized *β* = −0.27) and V_T_ (*β* = −0.03, 95% CI [−0.05, −0.01], *p* = 0.004; standardized *β* = −0.25), but not SampEn Bf (*β* = 0.00, 95% CI [−0.02, 0.02], *p* = 0.9).

#### 3.4.1 Cardiorespiratory fitness and ventilatory entropy

In secondary analyses, we found a non‐linear inverse relationship between Cardiorespiratory fitness (CRF) and SampEn measures (*p* < 0.001 for all models; Table S5). A higher V̇O_2peak_ was associated with lower SampEn (V̇_E_, V_T_, and Bf) values (Table S6). In models adjusted for age and BMI, V̇O_2peak_ was the primary determinant for SampEn V̇_E_ and Bf.

## 4 DISCUSSION

In a large cohort of healthy adults, we found differences in the ventilatory response to exercise across the age‐span. Specifically, older age was associated with a progressive shift towards ventilatory performance being achieved by a proportionally greater breathing frequency and heightened levels of ventilatory irregularity. The latter became most apparent after the sixth decade.

In health, the respiratory system adjusts minute ventilation in response to changes in metabolic rate and other perturbations, initially through increases in tidal volume, followed by hyperpnoea, with increasing exercise intensity (Tipton et al., 2017). The long‐studied impacts of healthy aging on the respiratory system demonstrate that it is associated with a loss of lung elastic recoil and reduced chest wall compliance providing an explanation for the preferentially tachypneic pattern seen in healthy aging (Janssens et al., 1999).

Our findings of an increase in ventilatory irregularity (described by SampEn V̇_E_) contrasts our hypothesis that aging would be associated with a decrease in this measure (i.e. as seen with heart rate variability (Umetani et al., 1998)). The application of entropy measures to evaluate respiratory physiology is limited. However, entropy has previously been used to characterize specific clinical scenarios, specifically, dysfunctional breathing and post‐COVID unexplained dyspnoea (Bansal et al., 2018; Samaranayake et al., 2023). In these conditions, individuals frequently report exertional breathlessness in the absence of any underlying cardiorespiratory pathology, and higher entropy values have been found compared to age‐matched healthy controls. An increase in entropy is postulated to arise from altered central respiratory drive or impaired integration of afferent feedback in the brainstem (Engoren, 1998). We speculate that the age‐related increase in entropy we found may reflect a similar alteration in central control. The observation that breathing frequency and ventilatory irregularity remain stable at the first ventilatory threshold suggests that at submaximal intensities, age‐related mechanical constraints (e.g., thorax stiffening and loss of lung elastic recoil) may not yet impair ventilation. However, as exercise intensity increases, a tachypneic‐dominant shift occurs potentially as a consequence of mechanical constraint or central dysregulation (or an interplay of both). Age‐related mechanical constraints result in longer inspiratory and expiratory durations, and changes to respiratory mechanics during exercise result in this shift towards a tachypneic pattern in older adults (Neder et al., 2003). The precise contribution of central dysregulation versus inefficient respiratory mechanics is not yet clear, and we acknowledge that this study does not have the data to resolve this directly. SampEn was used a descriptive marker rather than a mechanistic one, and the increase in entropy we observed is consistent with the broader notion of the loss of physiological complexity during aging (Manor & Lipsitz, 2013). Further investigation is needed to better understand these mechanisms.

The upward inflection in ventilatory SampEn occurs during the sixth decade, coinciding with the age at which exertional breathlessness becomes more prevalent in otherwise healthy individuals (Ramalho et al., 2019). Increases in irregularity may reflect less coordinated breathing control and an impaired ventilatory response to physiological stress compared to younger individuals, consistent with our findings. We provide normative values for ventilatory SampEn across the age span (Table 3), intended to serve as a reference standard for future studies.

Our findings are similar to previous studies evaluating the exercise ventilatory response in older adults. At peak exercise, we found a reduced tidal volume to forced vital capacity ratio, higher V̇_E_/V̇CO_2_ nadir, and a marked tachypneic shift when normalized for minute ventilation (Ofir et al., 2008). Together, these findings indicate that the increased ventilatory entropy in older adults reflects a system forced into a mechanically inefficient, frequency‐dominant pattern. Such neuromechanical dissociation may contribute to the heightened perception of breathlessness commonly reported with healthy aging. Breathing frequency is strongly associated with perceived exertion and breathlessness (Nicolò et al., 2016), and its stability across the age groups at the first ventilatory threshold further supports the notion that age‐related irregularity becomes most evident in higher intensity domains.

Our analysis revealed that males had higher SampEn for both minute ventilation and tidal volume. Females typically have smaller lung volumes and airway dimensions than height‐matched males (Bellemare et al., 2003), which may result in a reduced maximal ventilatory reserve and a greater propensity for expiratory flow limitation (Molgat‐Seon et al., 2018). It is plausible that this results in a narrower ventilatory response to exercise that can be characterized by a more regular time‐series.

To date, the application of SampEn to describe exercise ventilation has been limited. Blanks et al. (2024) reported declining gas exchange and ventilatory entropy values from early childhood through adolescence, with differences in SampEn oxygen uptake, minute ventilation and breathing frequency ranging from 5% to 40%. This suggests an increase in respiratory control during maturation. Similarly, Welch et al. (2026) recently assessed the differences between adolescents and young adults in ventilatory approximate during steady state exercise. They found higher entropy values in adolescent minute ventilation, breathing frequency and tidal volume compared to the adult group, further supporting the notion of an increase in respiratory control during maturation (Welch et al., 2026). These studies contrast with our findings of an increase in respiratory irregularity across the age‐span. Our findings may signal the loss of control during mid‐life and older age, consistent with the dedifferentiation of other physiological systems that is observed in frailty (Sleimen‐Malkoun et al., 2014). Taken together, these contrasting patterns across the age‐span indicate that changes in respiratory control may contribute to the response of ventilatory entropy during exercise, which likely reflects a stabilization and eventual decline of the response. However, Blanks et al. (2024), nor our study, measured central respiratory control, and these findings should be viewed as suggestive.

In our adjusted analysis, we found higher peak oxygen uptake values were associated with lower ventilatory SampEn. Although it appears the increase in entropy from 60‐years onwards aligns with the established decline in cardiorespiratory fitness that typically occurs after the fifth decade (Fleg et al., 2005), it is not yet clear if this finding is a statistical association or broader mechanistic relationship. Indeed, given the observational nature of the dataset, the association may reflect shared age‐related influences rather than a direct relationship between cardiorespiratory fitness and ventilatory irregularity. Nevertheless, future study is required to fully determine the relationship.

### 4.1 Methodological considerations

The primary limitation of this study is its retrospective, cross‐sectional design. To further study ventilatory irregularity within individuals over time and to explore the ventilatory response in aging, longitudinal data is needed. Furthermore, although our sample size is large, all testing was performed in a single center, from a single country and is predominantly Caucasian. This standardization supports internal consistency; however, it limits generalisability of these findings across different groups and populations.

An additional limitation of this analysis is the absence of perceptual breathlessness data (e.g., Borg scale), which precludes a direct correlation between entropy and symptom intensity in this cohort. While the observed tachypneic shift aligns with physiological patterns previously associated with perceived exertion, a future prospective study is required to explicitly define the relationship between ventilatory entropy and breathlessness.

We acknowledge that older adults exercised for shorter absolute durations, and at lower absolute intensities. This may have resulted in older adults spending less time at a high ventilatory demand and in a more regular (i.e. lower entropy) breathing pattern. However, our analysis found the proportion of the exercise test spent above the first ventilatory threshold was comparable across all age decades (52%–54%), suggesting that the relative distribution of exercise intensity was similar, regardless of age.

We acknowledge the use of an estimated rather than a directly measured MVV when calculating the percentage of breathing reserve. However, given the strong correlation between estimated and measured MVV in healthy individuals (American Thoracic Society, 2003), we considered this approach appropriate for the study.

In conclusion, this study provides new insight regarding the ventilatory response to vigorous exercise across the adult age‐span. Using entropy‐based analyses in a large cohort of healthy adults, we found that ventilatory irregularity increases in later life, particularly beyond the age of 60, and that aging is accompanied by a shift towards a breathing strategy characterized by higher breathing frequency and a constrained tidal volume response.

## AUTHOR CONTRIBUTIONS

**Zander J. Williams:** Visualization. **Giovanni Cenerini:** Formal analysis; visualization. **Fabian Schwendinger:** Data curation; formal analysis; visualization. **Raphael Knaier:** Data curation; formal analysis; investigation; methodology; project administration. **Jonathan Wagner:** Data curation; formal analysis; investigation; methodology; project administration. **James H. Hull:** Conceptualization; methodology; supervision; visualization. **Arno Schmidt‐Trucksäss:** Conceptualization; data curation; funding acquisition; investigation; methodology.

## FUNDING INFORMATION

G. Cenerini has received funding from the European Respiratory Society Clinical Training Fellowship 2024 for this work, and Z.J. Williams' salary is funded by the Royal Brompton and Harefield Hospital charity (Grant number: 153/C0124). Swiss National Science Foundation (182815). European Respiratory Society (ERS) (CTF202410‐01250).

## CONFLICT OF INTEREST STATEMENT

The authors declare no conflicts of interest.

## ETHICS STATEMENT

This study was approved by the Ethics Committee of Northwestern and Central Switzerland (EKNZ 2017–01451).

## Supporting information


**Table S1.** Participants characteristics by age decade.
**Table S2:** Multiple Linear Regression Predicting Ventilatory Reserve Utilization.
**Table S3:** Regression Models for Entropy Measures.
**Table S4:** Interaction of age and sex on entropy. Age was modeled using a penalized cubic spline.
**Table S5:** Multiple linear regression models of sample entropy versus V̇O_2peak_ (cardiorespiratory fitness).
**Table S6:** Physiological Determinants of Ventilatory Regularity (SampEn V̇_E_).

## Data Availability

Data will be made available upon reasonable request.
