# Supplementary material for: The ventilatory response pattern to exercise across the age‐span in healthy adults
Source: Physiol Rep. 2026 Sep 2;14(17):e71065. doi: 10.14814/phy2.71065 (PMC13539062; doi:10.14814/phy2.71065)
Supplement: Supplementary file 1 [file PHY2-14-e71065-s001.pdf]

THE VENTILATORY RESPONSE PATTERN TO EXERCISE ACROSS THE AGE-SPAN IN HEALTHY ADULTS

Zander J. Williams[1, †] and Giovanni Cenerini[1,2, †], Fabian Schwendinger[3,4], Raphael Knaier[3], Jonathan Wagner[3], James H. Hull[1,5*] and Arno Schmidt-Trucksäss[3,6*]

1. Department of Respiratory Medicine, Royal Brompton Hospital, London, UK

2. Department of Surgical, Medical and Molecular Pathology and Critical Care Medicine, University of Pisa, Pisa, Italy

3. Division of Sports and Exercise Medicine, Department of Sport, Exercise and Health, University of Basel, Basel, Switzerland

4. Division of Applied Exercise Physiology, Department of Sport, Exercise and Health, University of Basel, Basel, Switzerland

5. Institute of Sport, Exercise and Health (ISEH), Division of Surgery and Interventional Science, University College London, London, UK

6. Department of Clinical Research, University Hospital Basel, University of Basel, Basel, Switzerland

Corresponding Author: Prof. Arno Schmidt-Trucksäss; Arno.Schmidt-Trucksaess@unibas.ch

Division of Sport and Exercise Medicine, Department of Sport, Exercise and Health, University of Basel, Grosse Allee 6, CH-4052, Basel, Switzerland

Supplementary Table 1: Participants characteristics by age decade.

Characteristic	Overall (N=526)	20-29 (N=73)	30-39 (N=79)	40-49 (N=78)	50-59 (N=74)	60-69 (N=77)	70-79 (N=79)	80+ (N=66)
Age (years)	54 (20)	25 (3)	34 (3)	45 (3)	55 (3)	65 (3)	74 (2)	84 (3)
Females (%)	275 (52%)	40 (55%)	43 (54%)	38 (49%)	37 (50%)	41 (53%)	41 (52%)	35 (53%)
Body Mass Index (kg/m^2)	23.7 (2.7)	23.0 (2.8)	23.5 (2.4)	23.0 (2.7)	23.8 (2.5)	23.9 (2.5)	24.2 (2.9)	24.5 (3.1)
FEV_1 (L)	3.27 (0.93)	4.04 (0.82)	4.00 (0.78)	3.50 (0.55)	3.35 (0.74)	2.97 (0.71)	2.60 (0.68)	2.35 (0.65)
FEV_1 percent predicted	107 (22)	106 (18)	107 (19)	102 (18)	107 (21)	110 (21)	109 (26)	110 (28)
FVC (L)	4.19 (1.17)	4.98 (1.12)	4.99 (1.04)	4.55 (0.82)	4.30 (0.96)	3.91 (0.98)	3.47 (0.90)	3.04 (0.80)
FVC percent predicted	110 (22)	111 (21)	111 (20)	107 (20)	110 (21)	113 (23)	111 (26)	107 (24)
Values presented as Mean (SD) or n (%).								

Supplementary Table 2: Multiple Linear Regression Predicting Ventilatory Reserve Utilisation

Characteristic	Beta	95% CI	p-value
(Intercept)	14	[6.5, 22]	<0.001
Peak Breathing Frequency (breaths/min)	1.2	[1.0, 1.3]	<0.001
Age (years)	0.23	[0.17, 0.29]	<0.001
Values are presented as Beta [95% Confidence Interval]			

Supplementary Table 3: Regression Models for Entropy Measures.

	SampEn $\dot{V}_E$		SampEn V_T		SampEn Bf	
Characteristic	*Beta [95% CI]*	*p-value*	*Beta [95% CI]*	*p-value*	*Beta [95% CI]*	*p-value*
Age (non-linear)		0.01		0.04		0.023
Intercept	0.61 [0.17, 1.0]	0.006	2.3 [1.5, 3.1]	<0.001	1.5 [0.64, 2.3]	<0.001
Male	0.11 [0.03, 0.18]	0.004	0.05 [-0.08, 0.18]	0.5	0.09 [-0.05, 0.23]	0.2
BMI (kg/m^2)	0.00 [-0.01, 0.01]	0.5	-0.01 [-0.03, 0.01]	0.3	-0.01 [-0.02, 0.01]	0.6
$\dot{V}O_{2peak}$ (mL/kg/min)	-0.01 [-0.01, 0.00]	<0.001	-0.01 [-0.02, 0.00]	0.02	-0.01.00 [-0.02, -0.01]	<0.001
FEV1 (L)	0.04 [0.00, 0.09]	1.05	0.04 [-0.04, 0.12]	0.3	0.07 [-0.02, 0.15]	0.13
Exercise Duration (s)	-0.01 [-0.03, 0.00]	0.010	-0.03 [-0.05, -0.01]	0.04	0.00 [-0.02, 0.02]	0.9

Values are presented as Beta [95% Confidence Interval].

Supplementary Table 4: Interaction of age and sex on entropy. Age was modelled using a penalised cubic spline.

Characteristic	SampEn $\dot{V}_E$		SampEn V_T		SampEn Bf	
	Beta [95% CI]	p-value	Beta [95% CI]	p-value	Beta [95% CI]	p-value
Age (non-linear)		0.14		<0.001		0.4
Male	0.26 [-0.12, 0.64]		-0.16 [-0.83, 0.51]		0.34 [-0.38, 1.1]	
BMI (kg/m^2)	0.00 [-0.01, 0.01]	0.5	-0.01 [-0.03, 0.01]	0.2	-0.01 [-0.02, 0.01]	0.6
FEV_1(L)	0.04 [-0.01, 0.08]	0.13	0.04 [-0.04, 0.12]	0.3	0.06 [-0.02, 0.15]	0.2
$\dot{V}O_{2peak}$ (mL^{-1}/kg^{-1}/min)	-0.01 [-0.01, <0.001]	<0.001	-0.02 [-0.02, 0.00]	0.02	-0.01 [-0.02, <0.001]	<0.001
Exercise Duration (s)	-0.01 [-0.02, 0.00]	0.21	-0.03 [-0.05, -0.01]	0.06	0.00 [-0.02, 0.02]	>0.9
Age x Sex Interaction		0.017		0.004		0.6

Values are presented as Beta [95% Confidence Interval].

Supplementary Table 5: Multiple linear regression models of sample entropy versus $\dot{V}O_{2peak}$ (cardiorespiratory fitness).

Characteristic	SampEn $\dot{V}_E$	SampEn V_T	SampEn Bf
$\dot{V}O_{2peak}$ (mL/kg/min) [1]	-0.04 [-0.05, -0.04] ***	-0.04 [-0.06, -0.02] ***	-0.04 [-0.06, -0.02] ***
Age (years)	0.00 [0.00, 0.00]	-0.01 [-0.01, 0.00] ***	0.00 [0.00, 0.00]
BMI (kg/m^2)	0.00 [-0.01, 0.00]	-0.02 [-0.03, 0.00] *	-0.01 [-0.02, 0.01]

Values are presented as Beta [95% Confidence Interval]. [1]Peak VO_2 was modelled using a penalised cubic spline. *p < 0.05, **p < 0.01, ***p < 0.001.

Supplementary Table 6: Physiological Determinants of Ventilatory Regularity (SampEn $\dot{V}_E$).

Characteristic	Beta [95% CI]
(Intercept)	1.5 [0.95, 2.0] ***
Age (years)[1]	0.00 [0.00, 0.01]
Sex (Male vs. Female)	0.06 [-0.02, 0.13]
BMI (kg/m^2)	-0.01 [-0.01, 0.00]
$\dot{V}_E$ /VCO_2 Slope	0.00 [-0.01, 0.00]
FEV_1 (L)	0.04 [0.00, 0.08]
$\dot{V}O_{2peak}$ (mL/kg/min) [1]	-0.04 [-0.05, -0.03] ***

Values are presented as Beta [95% Confidence Interval]. [1] modelled using a penalised cubic spline with 4 knots. *$p < 0.05$, **$p < 0.01$, ***$p < 0.001$
